# Performance comparison of YOLO, Faster R-CNN, and HRNet architectures for bull sperm viability assessment

**DOI:** 10.3389/fvets.2026.1772252

**Published:** 2026-03-16

**Authors:** Ali Erdem Öztürk, Fatih İsmail Bahar, Yunus Emre Atay, Mustafa Bodu, Güven Güngör

**Affiliations:** 1 Department of Reproduction and Artificial Insemination, Faculty of Veterinary Medicine, Erciyes University, Kayseri, Türkiye; 2 ReproFellas Research Group, ArGePark Research Center, Erciyes University, Kayseri, Türkiye; 3 Faculty of Veterinary Medicine, Erciyes University, Kayseri, Türkiye; 4 Department of Obstetrics and Gynecology, Faculty of Veterinary Medicine, Erciyes University, Kayseri, Türkiye; 5 Department of Reproduction and Artificial Insemination, Faculty of Veterinary Medicine, Selçuk University, Konya, Türkiye; 6 Department of Veterinary Biostatistics, Faculty of Veterinary Medicine, Bingöl University, Bingol, Türkiye

**Keywords:** bull, Faster R-CNN, HRNet, sperm, viability, YOLO

## Abstract

Sperm viability analysis is directly related to fertility rates, and eosin/nigrosin staining is one of the most essential methods for assessing sperm viability. Although this method is easy to apply, it is time-consuming and has a high error rate due to its high subjectivity. Flow cytometric analyses, an alternative to the eosin/nigrosin method that is highly reliable, are not accessible due to the need for specialized personnel and high costs. Therefore, in this study, four different artificial intelligence models (YOLOv8, YOLOv12, Faster R-CNN, and YOLOv5 + HRNet) were utilized to analyze eosin/nigrosin-stained sperm samples with high reliability, and their performance was compared. For this purpose, 15 different frozen bull sperm samples were purchased, thawed in a water bath at 37 °C, and smears were prepared using the eosin/nigrosin method. A total of 3,068 photographs were taken from the prepared smears: 2140 for model training, 512 for validation, and 416 for testing. In the test set analysis, the YOLOv8 model demonstrated the highest overall performance (balanced accuracy: 97.1%; F1 score: 0.978), followed by YOLOv12 (balanced accuracy: 94.1%; F1 score: 0.958). While Faster R-CNN delivered mid-level performance, the YOLOv5 + HRNet model showed a significant performance loss, particularly in dead sperm detection, due to low specificity. To measure the clinical usability of the trained models, another smear set was prepared and photographed. In the independent mock test, 281 spermatozoa from 36 images were evaluated by the trained models and compared with both the ground truth and an expert evaluator. The expert achieved a balanced accuracy of 98.8%, while YOLOv12 demonstrated the highest performance among the AI models (balanced accuracy: 91.3%, F1 score: 0.895). Statistical comparison using McNemar’s test revealed no significant difference between YOLOv8, YOLOv12, Faster R-CNN, and the reference evaluation (*p* > 0.05). On average, AI-based analysis was 16.7 times faster than manual counting. The results reinforce the possibility that artificial intelligence models, updated with future studies, could be used in sperm viability analysis.

## 1 Introduction

Spermatological analyses are fundamental in andrology, as they determine the morphological, functional, and biochemical characteristics of spermatozoa (1). Information obtained about sperm is directly related to fertility and provides comprehensive information about an individual’s reproductive capacity (2). In veterinary medicine, spermatological parameters are of great importance in monitoring the reproductive performance of breeding animals (3). The data obtained from these analyses are particularly helpful in improving the effectiveness of assisted reproductive techniques such as artificial insemination and *in vitro* fertilization. By evaluating various functional and structural parameters of spermatozoa, it becomes possible to select suitable sperm samples for fertilization (4).

One of these analyses is sperm viability. Sperm viability analysis is a critical step in evaluating male infertility, and the eosin-nigrosin staining method is a commonly used traditional technique for this purpose (5). This method allows for the differentiation between live spermatozoa (unstained, appearing white) and dead spermatozoa (stained, appearing pink-red). Eosin stains the spermatozoa, while nigrosine stains the background, creating a distinct difference. Stained spermatozoa are prepared as smears and counted by an expert, with at least 300 specimens counted to provide the live/dead ratio as a percentage (6).

Although it is an easy, inexpensive, and non-specialized method, factors such as staining quality, microscope quality, and user experience significantly affect the results (7). At the same time, counting at least 300 spermatozoa in a smear and performing this procedure across multiple samples is a very slow, error-prone measurement method (8). As an alternative to conventional eosin-nigrosin analyses, flow cytometry has emerged over the past decade. Flow cytometry analysis relies on staining spermatozoa with fluorescent dyes such as SYBR-14/PI and performing live/dead analysis in a flow cytometer. In this analysis, samples are read in the flow cytometer after staining in the dark (9). Flow cytometry can distinguish between thousands of live and dead spermatozoa within a few minutes, providing highly reliable results with low subjectivity (10).

However, this method also has limitations. Flow cytometry devices are quite expensive and are not available in every laboratory. At the same time, it is necessary to have an expert operator with knowledge of the instruments and software to enable tabulation of the obtained data (11, 12). For this reason, researchers who own or have access to flow cytometry devices use subjective methods and obtain results with lower reliability than those from flow cytometric analyses.

In this case, both the eosin-nigrosin staining method and the flow cytometry method have their own disadvantages, and the need for an alternative method has become apparent. The main features that this method should include are: it should be inexpensive and easy to implement, capable of counting a large number of spermatozoa quickly and simultaneously, and highly accurate. To develop a solution to this dilemma, this study conducted a viability analysis using artificial intelligence.

In this study, smears prepared with eosin/nigrosin were trained to the machine using the YOLOv8, YOLOv12, Faster R-CNN, and YOLO v5 + HRNet frameworks. The study aimed to develop a method that is as simple and cost-effective as the traditional eosin/nigrosin technique, but capable of counting as many spermatozoa as flow cytometry and maintaining reliability.

## 2 Materials and methods

**Figure 1:**
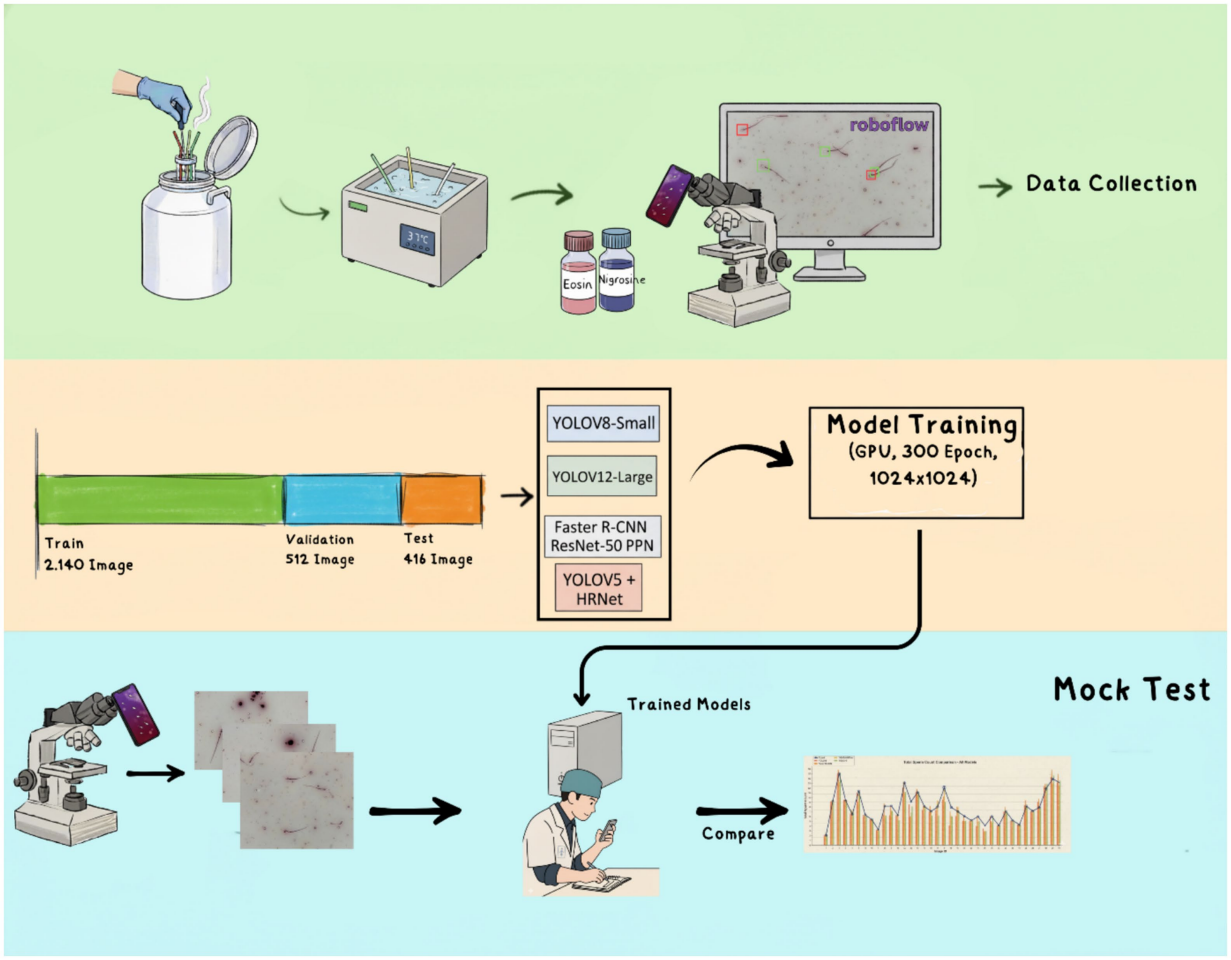
The first phase of the study involves sperm preparation and data collection (green background), the second phase includes dividing the data into training, validation, and test sets and training artificial intelligence models (orange background), and the third phase consists of comparing the artificial intelligence models with experts in real time (blue background).

Before a detailed explanation, the method used is summarized in Figure 1. The study’s workflow consists of sperm thawing and sample preparation with eosin–nigrosin at the first stage, followed by data collection via photographs and labeling. The second step involves classifying images into training, validation, and test categories and training the artificial intelligence models. The third stage consists of mock test results in which artificial intelligence models are compared with experts using the data obtained (Figure 1).

### 2.1 Preparation of the data set

First, the eosin-nigrosin solution was prepared as described by Björndahl et al. (13) and stored at 4 °C. The study used 15 bull semen straws stored frozen in liquid nitrogen. Each straw was dissolved in a 38 °C water bath for 25 s, and 1 drop of sperm was placed on a slide. Two drops of eosin-nigrosin stain were added to the sperm, and smears were prepared using a coverslip (24 × 24) (14). Three smears were taken from each sample, yielding a total of 30 smears for the experiment. Semen samples from 9 bulls were first processed and imaged, then allocated to the training dataset, whereas samples from the remaining 6 bulls were processed in later sessions and reserved for validation and test datasets.

**Figure 2:**
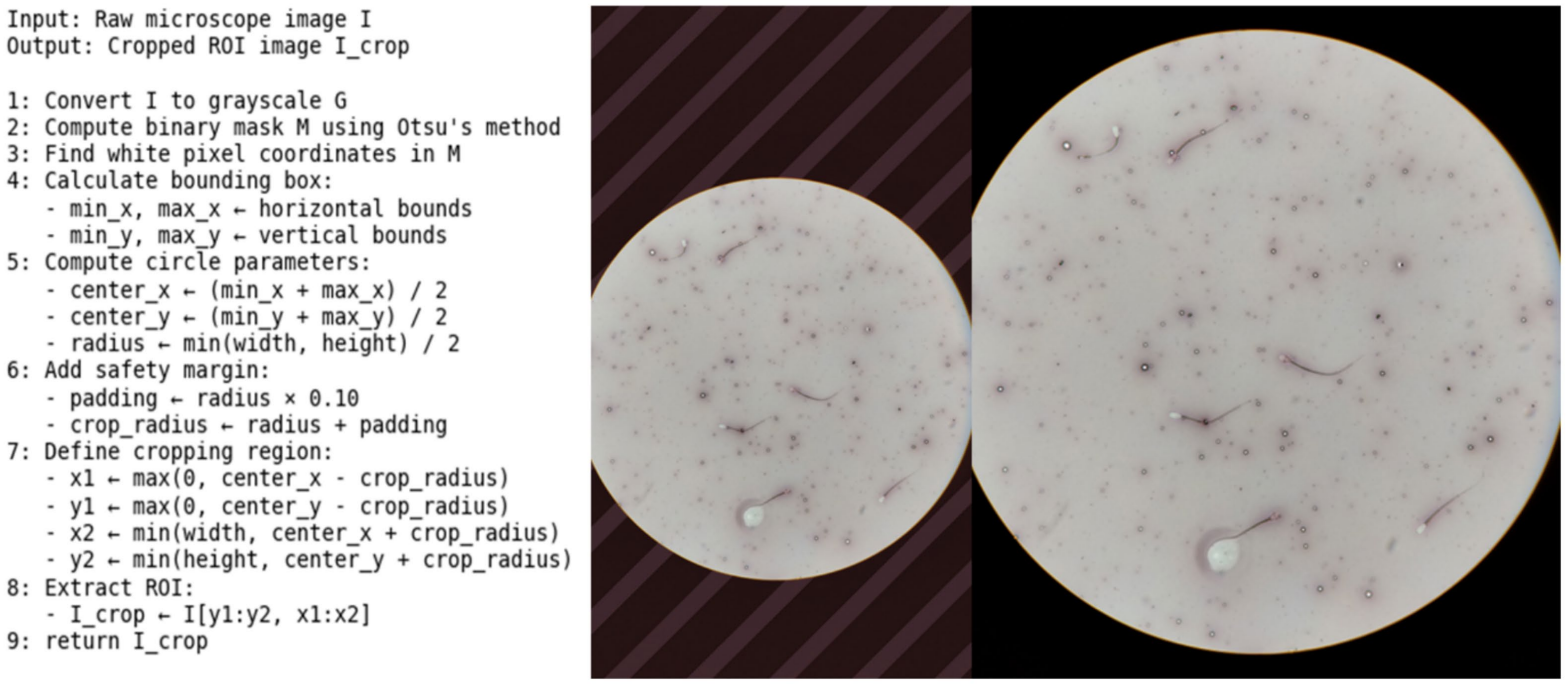
Cropping of microscopic images using the Otsu algorithm and preparation for annotation.

During the data collection phase, photographs were taken at 40x magnification using a light microscope (Zeiss, Primo Star, Germany) located in the Reproduction and Artificial Insemination Laboratory at Erciyes University. Photographing was performed using a mobile phone camera (iPhone, Apple, United States) for ease of access in field conditions and laboratory environments. A total of 3,068 microscopic images were obtained from 30 preparations. Of these, 2,140 images originated from the 9 bulls assigned to the training set, while the remaining 928 images were obtained from the 6 bulls reserved for validation and test datasets. To ensure uniformity in the raw microscope images, the background was cropped using the Otsu thresholding method, and the excess black area was removed (Figure 2). This pre-processing step was applied to 2,140 images prior to training and to 928 images prior to validation and testing.

Following the preprocessing stage, the dataset was divided into three subsets: 69% for training (2,140 images), 17% for validation (512 images), and 14% for testing (416 images) (15). The training set was used to train the model, the validation set to monitor performance during training, and the test set to evaluate the model’s generalization. When models perform well on training data but fail on new data, this is called overfitting and stems from the model memorizing the noise in the training data (16). Keeping the test set completely separate from the training process ensures that the model’s generalization error is estimated impartially (17). Therefore, the four trained models were evaluated on a test dataset not used during training.

**Figure 3:**
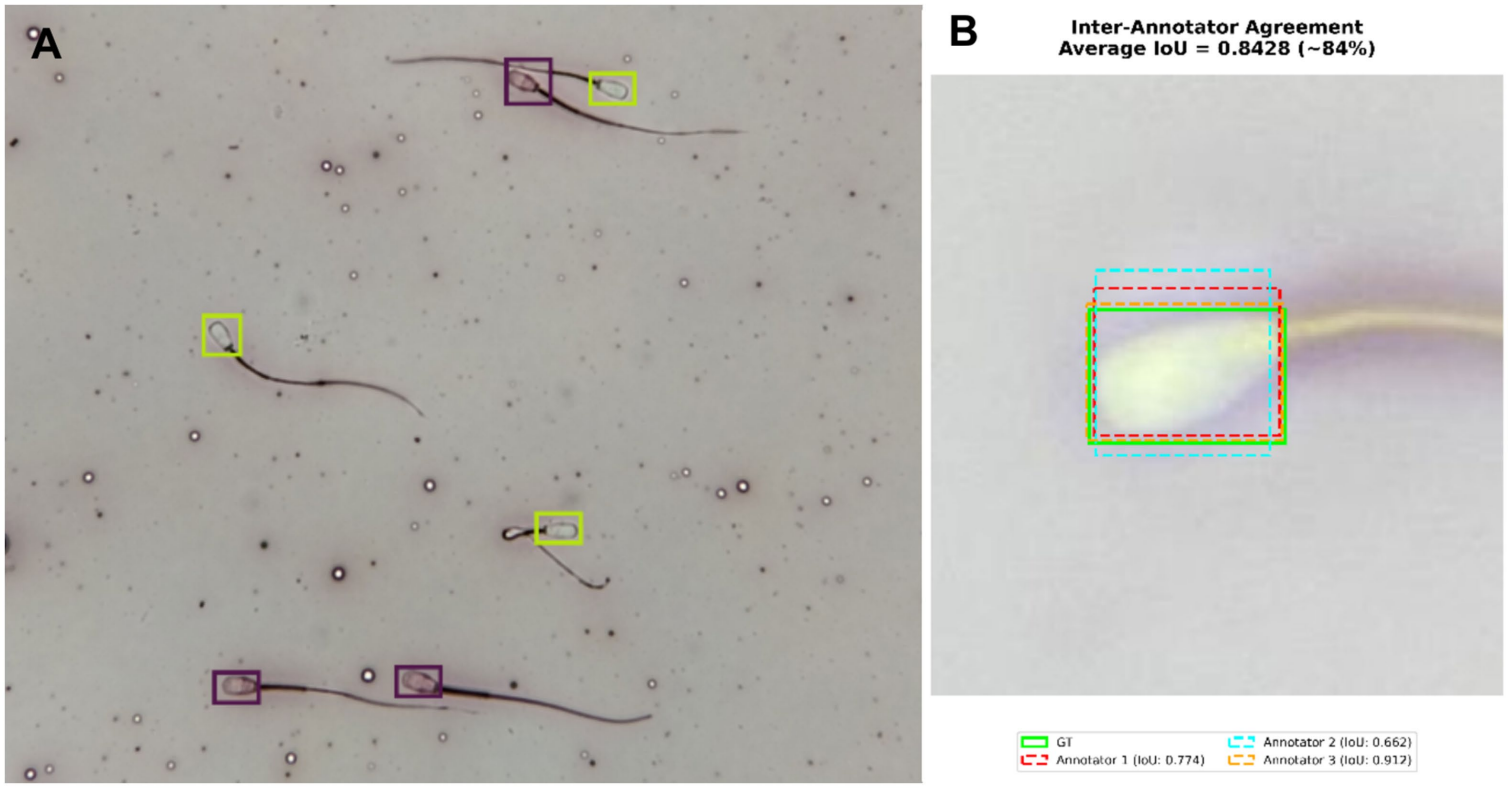
**(A)** Annotation of dead (purple box) and live (green box) spermatozoa using the Roboflow software. **(B)** Bounding box spatial consistency measured using the IoU metric.

A group of three professionals working in veterinary reproduction was formed to label images in the split dataset, and the labeled images were reviewed by two expert reviewers. The labeling process was carried out on the Roboflow platform, which enabled team members to collaborate and easily export data in the desired format. Images were annotated with bounding boxes to encompass the heads of each spermatozoon (Figure 3) (18). The labeling process for dead and live spermatozoa was carried out in accordance with the sperm viability assessment rules established by the World Health Organisation (WHO) based on the eosin-nigrosin staining method (19). According to these guidelines, only the heads of the spermatozoa were evaluated, with pink/red areas considered dead and white areas considered alive. However, spermatozoa that overlapped, had edges that appeared half-exposed, or had tails that passed over the head were not labeled, as they could cause confusion during training (20).

To evaluate labeling consistency among the annotators, 90 images were photographed from three smears prepared at different time points and not included in the dataset of this study, and a total of 1,000 spermatozoa (681 live, 319 dead) in these images were independently labeled by all three annotators. Inter-annotator agreement was quantitatively assessed at two different levels. First, the positional consistency of the bounding boxes was measured using the Intersection over Union (IoU) metric, widely used in object detection studies, yielding a mean IoU of 0.84 (Figure 3) (21).

$$\mathrm{IoU}=\frac{\left(A\cap B\right)}{\left(A\cup B\right)}$$

Secondly, Fleiss’ Kappa method was used to measure the consistency of class labeling among the three annotators (22). In the Fleiss’ Kappa coefficient, $P_{o}\;$ represents the observed proportion of actual agreement among the annotators, whereas $P_{e}\;$ represents the proportion of agreement expected purely by chance, taking class distributions into account. Among the 681 live spermatozoa included in the Fleiss’ Kappa analysis, 652 were labeled as live by all three annotators (complete agreement), and among the 319 dead spermatozoa, complete agreement was achieved in 309 cases. Partial agreement, defined as misclassification by one annotator, was observed in 39 spermatozoa. Under these conditions, the inter-annotator agreement measured by Fleiss’ Kappa was calculated as 0.94. According to the Landis and Koch (23) criteria, this value falls within the “almost perfect agreement” category (*κ* > 0.80), indicating a high level of consistency among the three expert annotators in spermatozoa classification.

$$\kappa =\frac{\left(\mathrm{Po}-\mathrm{Pe}\right)}{\left(1-\mathrm{Pe}\right)}$$

As a result of labeling, 7,340 dead and 13,023 live spermatozoa were detected in the training set, 1,310 dead and 2,704 live spermatozoa in the validation set, and 1,008 dead and 2,140 live spermatozoa in the test set. The distribution of sperm counts across the training, validation, and test sets is summarized in Table 1. Class balancing techniques were not applied because the observed class distribution (approximately 1.8:1; ≈65% live/35% dead) falls below the 2:1 imbalance threshold. According to He and Garcia (24), imbalance ratios below 2:1 are generally considered mild and may not require specific balancing strategies. During export, all images were scaled to a fixed size of 1,024 × 1,024 pixels and exported in the Common Objects in Context (COCO) format.

**Table 1:** The number of total labeled spermatozoa according to the training set, validation set, and test set, and their ratios in terms of dead and live spermatozoa.

Metrics	Image count	Dead-head	Live-head	Total sperm
Train set	2,140(70%)	7,340 (36.0%)	13,023 (64.0%)	20,363
Validation set	512 (17%)	1,310 (32.6%)	2,704 (67.4%)	4,014
Test set	416 (14%)	1,008 (32.0%)	2,140 (68.0%)	3,148
Total	3,068 (100%)	9,658 (35.1%)	17,867 (64.9%)	27,525

### 2.2 Selection of AI models

In this study, the YOLOv8 Small (25), YOLOv12 (26), Faster R-CNN (27), and YOLOv5 + HRNet (22) architectures were employed. YOLOv8 Small provides precise detection of small objects thanks to its anchor-free structure and decoupled head design. Unlike the standard YOLOv8, the architecture is optimized for small spermatozoa by using only the P3 level (8x downsampling). By removing the P4 and P5 levels from the architecture, computational costs are reduced and detection power is increased. Feature extraction has been enhanced by using C2f modules in the backbone, and features at different scales have been integrated using the SPPF module. A single-level detection strategy has been applied in the detection head to enable high-resolution processing of small objects. The model optimizes localization accuracy by combining binary cross-entropy, Complete IoU, and Distributional Focal Loss (DFL) (28–30).

YOLOv12 achieves high accuracy and speed by leveraging an attention-mechanism-centric architecture, unlike previous CNN-based YOLO versions. The model’s key innovation is the Area Attention (*A*^2^) mechanism. This mechanism significantly reduces computational costs while preserving the wide receiver area by dividing feature maps into horizontal or vertical regions. The Residual Efficient Layer Aggregation Networks (R-ELAN) module used in the backbone improves feature aggregation, enhancing the ability to detect small and thin spermatozoa (26). YOLOv12 increases model speed by addressing the memory-access bottleneck via FlashAttention integration. The MLP ratio is set to 1.2 to balance computation between attention and the forward feed network, and spatial information is implicitly encoded via 7 × 7 separable convolutions. The model’s anchor-free design and decoupled heads structure independently optimize object detection, classification, and location regression tasks, thereby improving overall performance (26).

Faster R-CNN achieves high accuracy thanks to its two-stage architecture. In the first stage, the Region Proposal Network (RPN) proposes potential object regions, and in the second stage, these regions are classified to perform detection. ResNet-50, used as the backbone, performs effective feature extraction in deep layers thanks to its connections. Multi-scale feature pyramids have been generated using the Feature Pyramid Network (FPN). FPN’s top-down path and lateral connections combine high-level semantic information with low-level spatial information, enhancing the detection of spermatozoa of different sizes (27).

The YOLOv5 + HRNet architecture combines HRNet’s high-resolution feature extraction capability with YOLOv5’s detection mechanism. The HRNet backbone produces feature maps at resolutions of 1/4, 1/8, 1/16, and 1/32 through 4 parallel streams. Each stream contains residual units and 3 × 3 convolutions. Unlike ResNet, the high-resolution stream is preserved from start to finish, and continuous information exchange is ensured between parallel streams. Fusion modules are applied every 4 residual units to combine representations at different resolutions. The multi-resolution features obtained from HRNet are fed into the FPN and PAN structures to create a multi-scale feature pyramid for detecting spermatozoa at different scales. The detection process is performed at three different scales using an anchor-based approach (31).

### 2.3 Hyperparameters

Hyperparameters are fundamental factors that govern the training process of machine learning models and directly influence model performance. The hyperparameters used in this study were determined based on standard values widely accepted in the literature (26). For YOLO-based models (YOLOv8 Small, YOLOv12, and YOLOv5 + HRNet), the default hyperparameter configurations recommended by Ultralytics were used, while for Faster R-CNN, the standard settings of the Detectron2 framework were applied (32). The image size was fixed at 1024 × 1,024 for all models, and this resolution value was maintained during both the training and testing phases. In YOLO models, the learning rate was set to 0.01 and the momentum to 0.937. A warmup strategy was applied during the first 3 epochs of training to ensure the model began learning in a stable manner (33, 34). Data diversity was increased using data augmentation techniques such as horizontal flip (0.5 probability), mosaic augmentation (1.0), and HSV color space manipulations (H: 0.015, S: 0.7, V: 0.4). The box loss weight in the loss function was set to a range of 0.05–7.5, while the class loss weight was set to 0.5. The patience parameter was set to 50 epochs, triggering early stopping (Table 2).

**Table 2:** YOLO based models training hyperparameters.

Category	Hyperparameter	Value(s)
Basic Training Parameters	Epoch	300
	Batch Size	16
	Image Size	1,024
	Patience	50
	Workers	6–8
Optimizer Parameters	Base Learning Rate (lr0)	0.01
	Final LR Factor (lrf)	0.01
	Weight Decay	0.0005
	Momentum	0.937
	Warmup Epochs	3.0
	Warmup Momentum	0.8
Loss Parameters	Box Loss Weight	0.005–7.5
	Class Loss Weight	0.5
Augmentation Parameters	Horizontal Flip	0.5
	Mosaic	1.0
	Mixup	0.0
	HSV-H	0.015
	HSV-S	0.7
	HSV-V	0.4
	Scale	0.5
	Translate	0.1

### 2.4 Training of models

Since YOLO and Faster R-CNN models have different architectures, they use different loss functions during training. YOLO models, as single-stage detectors, optimize box loss, class loss, and DFL components, whereas Faster R-CNN, as a two-stage detector, computes total loss, classification loss, and box regression loss. During training, performance was checked on the validation set at the end of each epoch, and the model weights with the highest mean average precision 50–95 (mAP50-95) value were saved. The patience = 50 parameter was used in both architectures, and training was automatically terminated if no improvement was observed over 50 epochs (16). This reduces overfitting to some extent and captures the point at which the model performs best.

**Figure 4:**
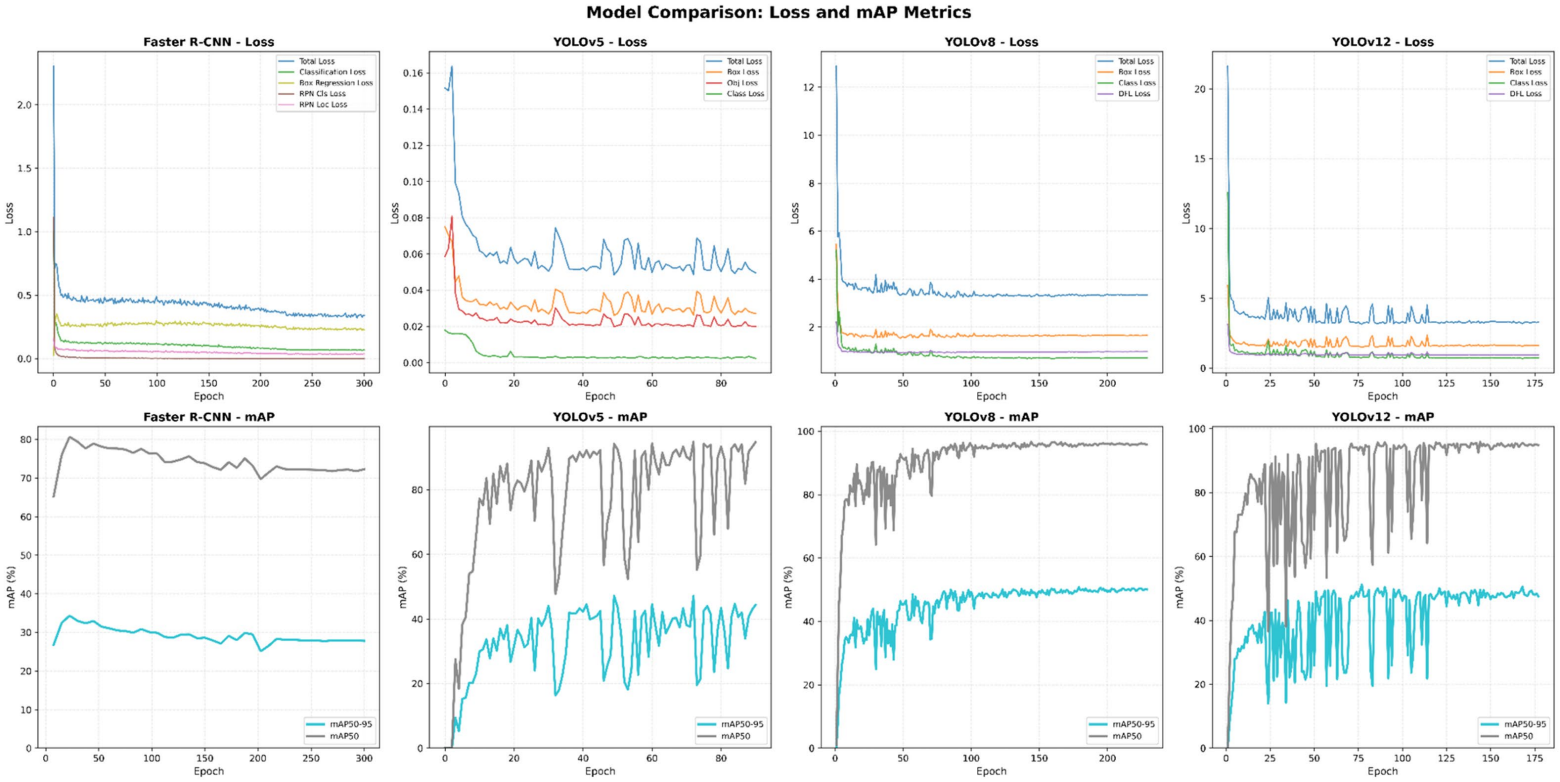
Training performance of the models over 300 epochs. Top panel for each model: decrease in loss functions (box, class, DFL loss). Bottom panel: development of accuracy metrics (mAP50-95 and mAP50).

The models were trained for 300 epochs using an A100 GPU in the Google Colab environment. The YOLOv12 model completed training in 5.5 h, the YOLOv8-small model in 5.2 h, the Faster R-CNN model in 6.9 h, and the YOLOv5 + HRNet model in 6.2 h. The YOLOv12 model reached its highest accuracy at epoch 77, the YOLOv8 model at epoch 179, the YOLOv5 + HRNet model at epoch 49, and the Faster R-CNN model at epoch 133. The training times of the models and the epoch values at which they achieved their highest accuracy are shown in Table 3, while the loss functions and changes in mAP50-95 accuracy throughout the epochs are shown in Figure 4. The model weights obtained in the epochs with the best performance (highest mAP50-95) were recorded and used in the testing phase.

**Table 3:** Training durations of the models, epoch values at which they achieved the highest accuracy, and mAP50-95 performance results.

Model	Training time	Optimum epoch	Highest mAP50-95 (%)
YOLOv12	5.5 h	77	51.21
YOLOv8	5.2 h	179	50.80
YOLOv5-HrNet	6.2 h	49	47.16
Faster R-CNN	6.9 h	22	34.34

### 2.5 Classification metrics

For each model, a confusion matrix for binary classification was constructed by comparing the predicted class labels with the ground-truth labels (Table 4). In this matrix, true positives (TP) represent spermatozoa correctly assigned to their respective class; false positives (FP) denote spermatozoa incorrectly classified or non-sperm objects mistakenly detected as spermatozoa; false negatives (FN) indicate spermatozoa that were present but either not detected or incorrectly classified; and true negatives (TN) correspond to correctly rejected instances.

**Table 4:** Confusion matrix example.

Model Prediction		Reference (ground truth)	
		Live	Death
Model	Live	True Positive (TP)	False Positive (FP)
	Death	False Negative (FN)	True Negative (TN)

Based on these definitions, several classification performance metrics were calculated (35). Recall (Sensitivity) represents the proportion of spermatozoa that truly exist and are correctly detected by the model and was calculated as follows:

$$\text{Recall}=\frac{\mathrm{TP}}{\mathrm{TP}+\mathrm{FN}}$$

Specificity indicates the proportion of negative cases that are correctly identified and was defined as:

$$\text{Specificity}=\frac{\mathrm{TN}}{\mathrm{TN}+\mathrm{FP}}$$

Overall accuracy represents the proportion of correctly classified instances among all predictions and was calculated as:

$$\text{Accuracy}=\frac{\mathrm{TP}+\mathrm{TN}}{TP+\mathrm{TN}+\mathrm{FP}+\mathrm{FN}}$$

To reduce the effect of potential class imbalance, Balanced Accuracy was calculated as:

$$\text{Balanced Accuracy}=\frac{\text{Sensitivity}+\text{Specificity}}{2}$$

Precision (Positive Predictive Value, PPV) represents the proportion of instances predicted as positive that are truly positive and was calculated as:

$$\text{Precision}=\frac{\mathrm{TP}}{\mathrm{TP}+\mathrm{FP}}$$

Negative Predictive Value (NPV) represents the proportion of instances predicted as negative that are truly negative:

$$\mathrm{NPV}=\frac{\mathrm{TN}}{\mathrm{TN}+\mathrm{FN}}$$

The F1-score, defined as the harmonic mean of Precision and Recall, evaluates the balanced performance of the model and was calculated as:

$$F1=\frac{2\times (\text{Precision}\times \text{Recall})\;}{\text{Precision}+\text{Recall}}$$

To assess the overall discriminative ability of the model, the area under the receiver operating characteristic curve (AUC-ROC) was computed. The ROC curve is based on the relationship between the true positive rate (TPR) and the false positive rate (FPR):

$$\mathrm{TPR}=\frac{\mathrm{TP}}{\mathrm{TP}+\mathrm{FN}}$$

$$\mathrm{FPR}=\frac{\mathrm{FP}}{\mathrm{FP}+\mathrm{TN}}$$

**Figure 5:**
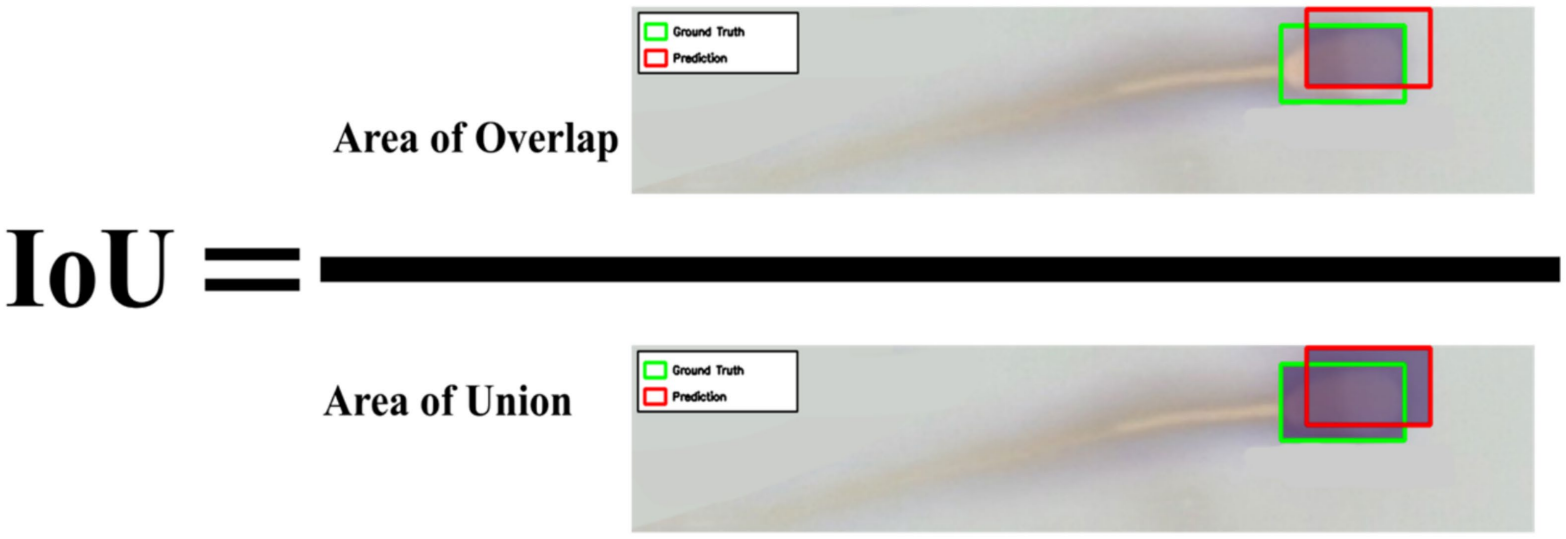
Calculation of the IoU ratio. The area of overlap represents the intersection between the actual label and the machine prediction, while the area of union represents the union of both.

For evaluating object detection performance, Intersection over Union (IoU) and mean Average Precision (mAP) were used. IoU measures the overlap between the predicted and ground-truth bounding boxes (Figure 5). mAP50 denotes the average precision when the IoU threshold is set to 50%. For a more rigorous evaluation, mAP50-95—the average mAP value obtained by varying the IoU threshold from 50 to 95% in steps of 5% was used (36). This metric provides a more comprehensive accuracy measurement by evaluating the model’s overall performance at various sensitivity levels and is used as the standard for evaluating the COCO dataset (27). All metrics were calculated separately for the live and dead sperm classes (37).

### 2.6 Mock test

To evaluate the performance of the models in a real-world scenario, an independent mock test was conducted separate from the training and test sets. A slide was prepared, stained with eosin-nigrosin, and photographed 36 times with a standard-resolution camera. The images were labeled by two experts after being checked three times (total of 281 spermatozoa: 171 dead, 110 alive). A different expert counted the same images subjectively using the conventional method, and the time taken was recorded. The trained models were tested on the same images and compared with the expert performance (time taken and accuracy rate).

### 2.7 Statistical analysis

Additional analyses were conducted to ensure the reliability of performance metrics and to establish a statistical basis for model comparisons. The 95% confidence intervals for all proportional performance metrics (Recall, Specificity, Accuracy, etc.) were calculated to indicate estimation precision and generalizability. The chance-corrected agreement between model predictions and expert evaluation (ground truth) was measured using Cohen’s Kappa (*κ*) statistic. The Youden Index (J = Sensitivity + Specificity − 1) was computed to summarize the model’s overall discriminative power in a single value. McNemar’s test was used to assess the statistical significance of differences in performance rates between two classifiers evaluated on the same dataset (e.g., a model versus an expert, or two different models). To statistically compare the overall discriminative ability of the models, DeLong’s test was performed on the Area Under the Curve (AUC) values derived from Receiver Operating Characteristic (ROC) analysis. This non-parametric test evaluates whether the difference between two correlated AUCs is statistically significant, accounting for the paired nature of the data. All statistical calculations and tests were performed using R 4.3.2 software.

## 3 Results

### 3.1 Test set performance

According to the test set results (Table 5), the YOLO v8 model achieved the highest mAP50 score for detecting dead spermatozoa (0.9772). In contrast, YOLO v12 yielded the best mAP50 result for detecting live spermatozoa (0.9856). When comparing the total (average) mAP50 values, YOLO v8 was the most successful model overall with a score of 0.9799. The Faster R-CNN model lagged behind YOLO models, with a total mAP50 of 0.7543, while YOLO v5 + HRNet achieved the lowest total mAP50 at 0.6797. However, YOLO v5 + HRNet’s high mAP50 for live spermatozoa (0.9324) is misleading. As evidenced by the confusion matrix in Table 6, this model incorrectly classified a very high number of dead spermatozoa as live [FP: 786 (25%)]. This high rate of false live detections artificially inflates its mAP50 score for the live class but results in poor performance for the dead class (0.4270 mAP50) and a low overall average. The performance of all models decreased significantly in the stricter mAP50-95 metric, with YOLO v8 and v12 showing comparable and superior overall performance (0.5684 and 0.5727 total mAP50-95, respectively).

**Table 5:** Performance of models in detecting dead and live sperm in the test set using mAP50 and mAP50-95.

Validation metrics	Class	YOLOv8	YOLOv12	Faster R-CNN	YOLO v5 + HRNet
mAP50	Dead Sptz.	**0.9772**	0.9523	0.7254	0.4270
	Live Sptz.	0.9825	**0.9856**	0.7832	0.9324
	Total	**0.9799**	0.9690	0.7543	0.6797
mAP50-95	Dead Sptz.	0.5619	0.5552	0.3025	0.1767
	Live Sptz.	0.5748	0.5902	0.3536	0.4573
	Total	0.5684	**0.5727**	0.3281	0.3170

Highest mAP50 and mAP50-95 values are written in bold.

**Table 6:** Confusion matrices for YOLOv8, YOLOv12, Faster R-CNN, and YOLOv5-HRNet on test sets for detecting live and dead spermatozoa.

Model	Class	Reference (ground truth)		Total
		Live	Death	
YOLO v8	Live	2076 (66.0%)	29 (0.9%)	
	Death	64 (2.0%)	979 (31.1%)	
	Total	2,140 (68.0%)	1,008 (32.0%)	3,148 (100%)
YOLO v12	Live	2025 (64.3%)	64 (2.0%)	
	Death	115 (3.7%)	944 (30.0%)	
	Total	2,140 (68.0%)	1,008 (32.0%)	3,148 (100%)
Faster R-CNN	Live	1959 (62.2%)	188 (6.0%)	
	Death	181 (5.7%)	820 (26.1%)	
	Total	2,140 (68.0%)	1,008 (32.0%)	3,148 (100%)
YOLOv5 HRNet	Live	1983 (63.0%)	786 (25.0%)	
	Death	157 (5.0%)	222 (7.0%)	
	Total	2,140 (68.0%)	1,008 (32.0%)	3,148 (100%)

When examining the overall localization and classification accuracy under stricter criteria (mAP50-95), YOLOv12 performed best with a score of 0.5727, closely followed by YOLOv8 at 0.5684. The performance of Faster R-CNN (0.3281) and YOLOv5 + HRNet (0.3170) was significantly lower (Table 5). Notably, YOLOv5 + HRNet’s very low score for dead sperm detection (0.1767 mAP50-95) aligns with its confusion matrix (Table 6), which shows a critical failure to identify dead cells, with most (786 out of 1,008) being incorrectly predicted as live.

When evaluating the models’ performance in distinguishing between live and dead spermatozoa (with live sperm as the positive class), YOLOv8 demonstrated the highest overall performance on the test set (confusion matrix in Table 6, performance metrics in Table 7), with a balanced accuracy of 97.1% (95% CI: 96.4–97.7%). It achieved a recall (sensitivity) of 97.0% and a specificity of 97.1%, yielding an F1 score of 0.978. YOLOv12 showed slightly lower but still strong performance, with a balanced accuracy of 94.1% (95% CI: 93.2–95.0%), a recall of 94.6%, and an F1 score of 0.958.

**Table 7:** Performance comparison of YOLOv8, YOLOv12, Faster R-CNN, and YOLOv5-HRNet model test sets in detecting live and dead spermatozoa.

Metrics	Models			
	YOLOv8	YOLOv12	Faster R-CNN	YOLOv5 HRNet
Recall	0.970 (0.962–0.977)	0.946 (0.936–0.955)	0.915 (0.903–0.927)	0.923 (0.915–0.937)
Specificity	0.971 (0.959–0.981)	0.937 (0.920–0.951)	0.814 (0.788–0.837)	0.220 (0.195–0.247)
Accuracy	0.971 (0.964–0.976)	0.943 (0.935–0.951)	0.883 (0.871–0.894)	0.700 (0.684–0.716)
Balanced accuracy	0.971 (0.964–0.977)	0.941 (0.932–0.950)	0.864 (0.851–0.878)	0.573 (0.559–0.587)
Precision	0.986 (0.980–0.991)	0.969 (0.961–0.976)	0.912 (0.899–0.924)	0.716 (0.699–0.733)
NPV	0.939 (0.922–0.952)	0.891 (0.871–0.910)	0.819 (0.794–0.843)	0.586 (0.534–0.636)
F1 Score	0.978 (0.974–0.983)	0.958 (0.952–0.964)	0.914 (0.905–0.823)	0.808 (0.796–0.820)
AUC	0.971 (0.963–0.978)	0.941 (0.931–0.952)	0.864 (0.849–0.880)	0.573 (0.551–0.596)
IOU	0.791 (0.785–0.796)	0.804 (0.798–0.810)	0.733 (0.727–0.739)	0.762 (0.756–0.768)
Cohen’s Kappa	0.933 (0.919–0.946)	0.871 (0.852–0.889)	0.730 (0.705–0.756)	0.176 (0.143–0.209)
Youden Index	0.941 (0.929–0.954)	0.883 (0.865–0.901)	0.729 (0.702–0.756)	0.147 (0.119–0.175)
McNemar’s Test	*χ*^2^ = 13.17, df = 1, *p* < 0.001	*χ*^2^ = 14.53, df = 1, *p* < 0.001	*χ*^2^ = 0.13, df = 1, *p* = 0.716	*χ*^2^ = 419.56, df = 1, *p* < 0.001

NPV, negative predictive value; AUC, area under the curve; IOU, intersection over union; χ^2^, Chi-Square; df, degrees of freedom; *p*, *p*-value.

In contrast, Faster R-CNN showed a significant performance decline, with balanced accuracy of 86.4% (95% CI: 85.1–87.8%), recall of 91.5%, and specificity of 81.4%. The YOLOv5 + HRNet model performed the poorest, with a critically low specificity of 22.0% (95% CI: 19.5–24.7%) and a balanced accuracy of only 57.3% (95% CI: 55.9–58.7%). This indicates a severe deficiency in correctly identifying dead (negative) spermatozoa. Statistical tests further supported these findings (Table 7). McNemar’s test indicated a significant difference between the YOLO-based models’ predictions and the ground truth (*p* < 0.001). In contrast, the difference for Faster R-CNN was not statistically significant (*p* = 0.716). The very low Kappa statistic for YOLOv5 + HRNet (*κ* = 0.176) confirms its poor agreement with the ground truth.

**Figure 6:**
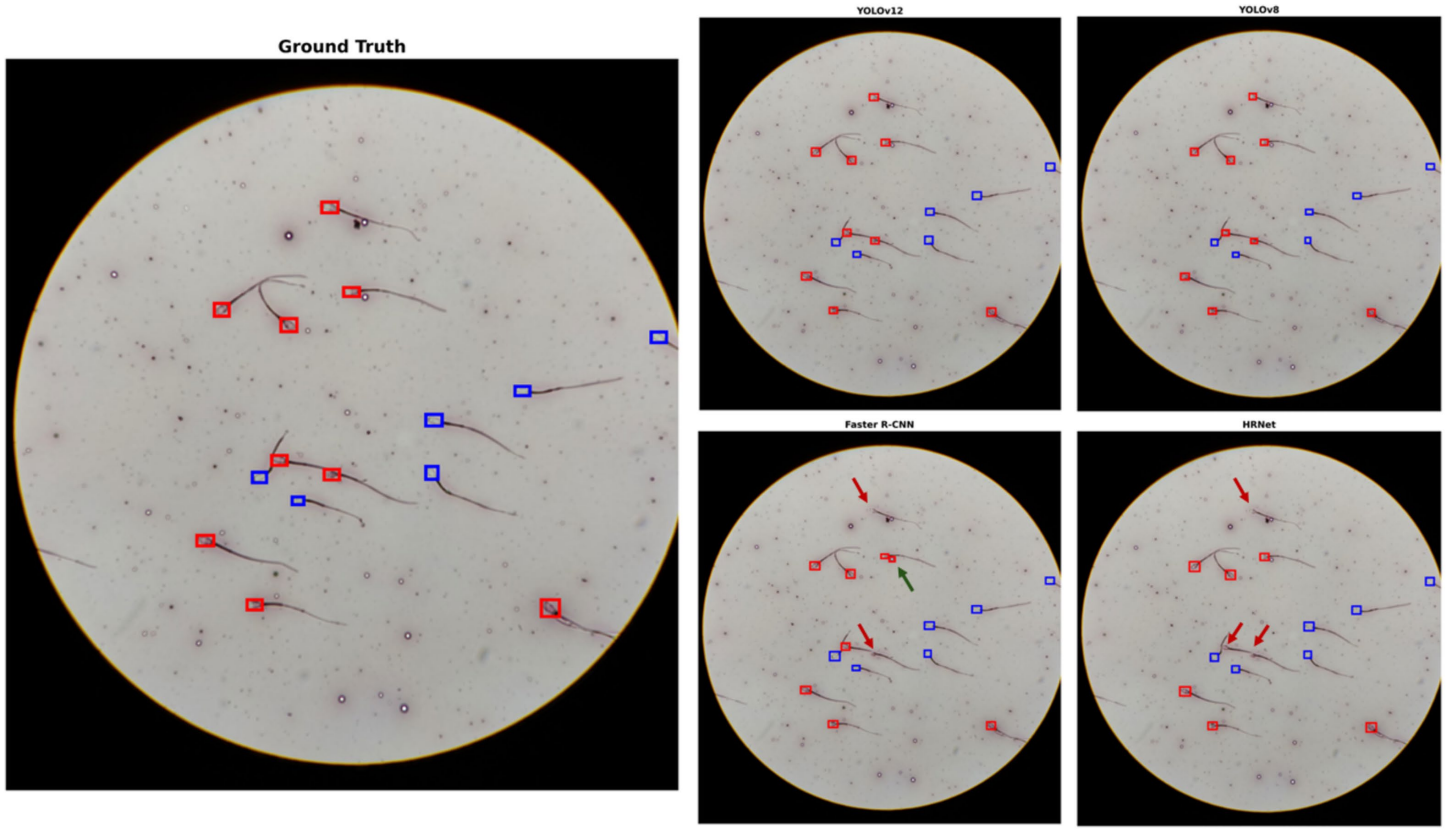
Predictions of YOLOv8, YOLOv12, Faster R-CNN, and YOLOv5-HRNet on the same test data. Red arrows indicate spermatozoa that were not detected (FN), while green arrows indicate predictions that were mistaken for spermatozoa despite not being spermatozoa (FP).

To better understand the quantitative results, a visual example from the test dataset is provided in Figure 6. The visual inspection aligns with the high performance metrics of YOLOv8 and YOLOv12 in Table 7, as their predictions in the image match the ground truth perfectly. In contrast, the visual findings explain the lower scores of the other two models. Faster R-CNN’s false positive (marking an air bubble as sperm) and false negative (missing two spermatozoa) observed in Figure 6 are consistent with its elevated FP and FN rates visible in the confusion matrix (Table 6) and its moderate performance metrics in Table 7 (e.g., Specificity: 0.814, Kappa: 0.730). Figure 6 provides a concrete visual example of YOLOv5 + HRNet’s critical failure: it missed three dead spermatozoa. Consequently, the model achieves the worst scores in key metrics in Table 7, most notably a Specificity of 0.220 and a Kappa of 0.176, indicating agreement barely better than chance.

Youden’s Index, which summarizes a model’s balanced discriminatory power, further confirms this performance hierarchy. YOLOv8 achieved the highest index (0.941), while YOLOv12 (0.883) and Faster R-CNN (0.729) scored progressively lower. The critically low index for YOLOv5 + HRNet (0.147) starkly quantifies its near-chance-level failure to correctly identify dead spermatozoa.

### 3.2 Mock test performance

**Figure 7:**
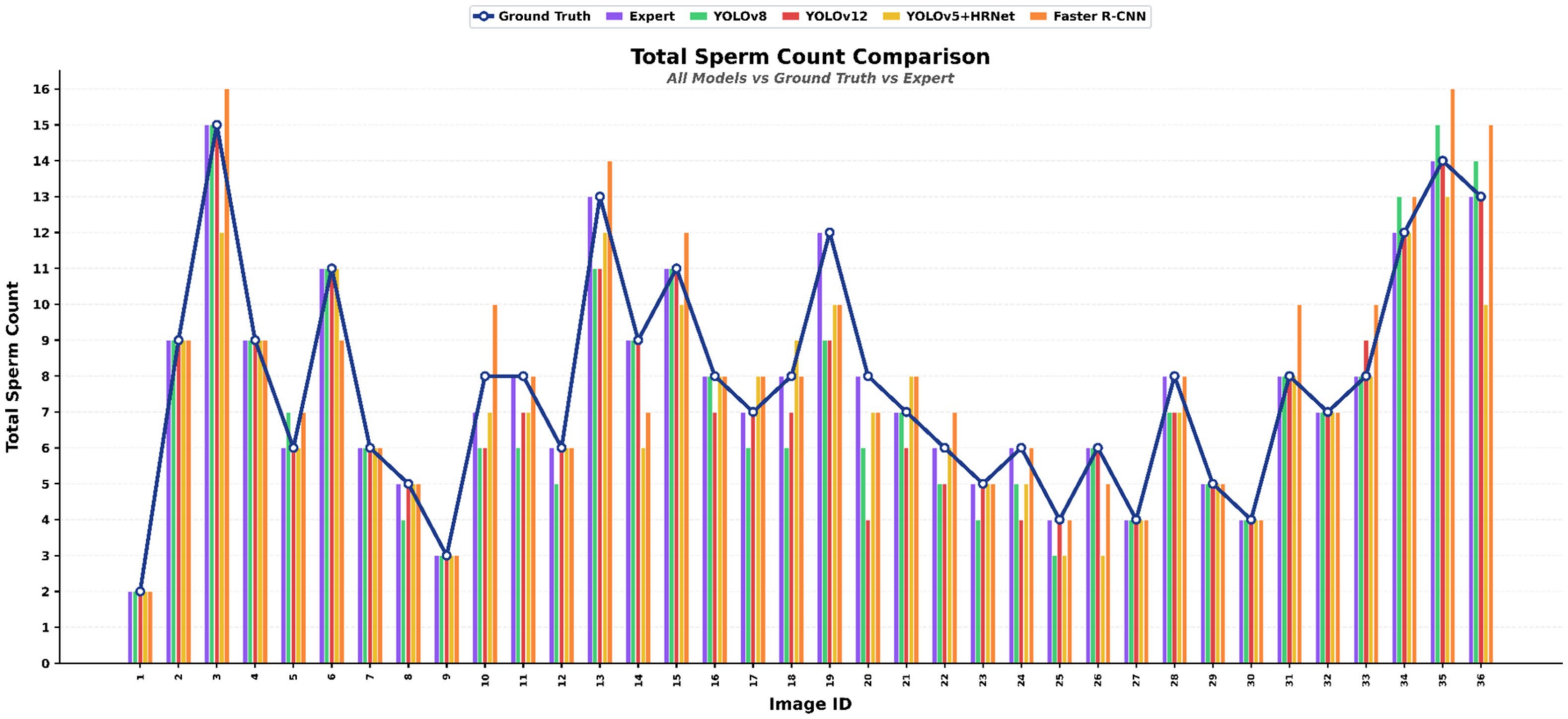
The accuracy rates of each model in correctly identifying the number of spermatozoa in each image. Ground Truth baseline (blue line), expert (purple), YOLOv12 (orange), Faster R-CNN (green), YOLOv5 HRNet (yellow), YOLOv8 (red).

**Figure 8:**
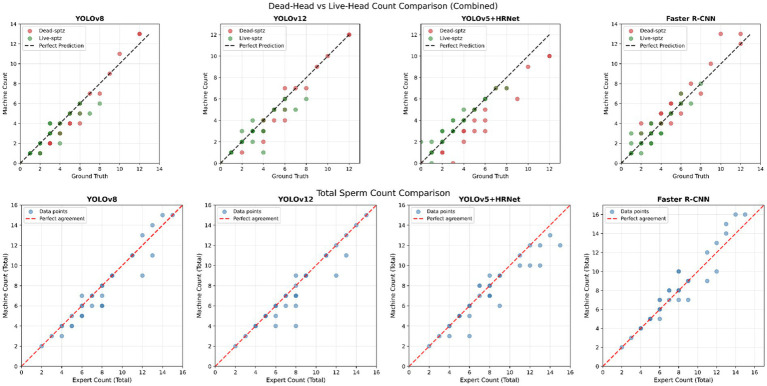
The accuracy rates of the models in correctly predicting the total spermatozoa count (bottom) and the number of dead and live spermatozoa (top) based on the spermatozoa density in the image. When the models correctly predict the spermatozoa count in the image or the number of dead and live spermatozoa, they are positioned on the middle line. Darker colored points represent multiple images containing the same number of spermatozoa.

A total of 36 images containing 281 spermatozoa (171 dead, 110 alive) in the mock test set were tested on trained models. The average detection time for models using a graphics processing unit (GPU) was measured at 18.75 s, while that for models using a central processing unit (CPU) was 59.19 s. Expert labeling took 5 min and 23 s. Accordingly, the models delivered results 16.7 times faster on the GPU and 5.3 times faster on the CPU than the expert. The mock test results were evaluated in two stages. The first was the estimated spermatozoa count per image (Figure 7), and the second was the accuracy of the spermatozoa count prediction based on the scarcity or abundance of spermatozoa in the image and the accuracy of the known spermatozoa count in terms of dead and live ratios (Figure 8).

Figure 7 compares the ground truth, the models, and the expert in terms of estimated spermatozoa counts per image. The expert made only one mistake in determining the spermatozoa count across all images; however, the artificial intelligence models produced results identical to the ground truth in some images but incorrect in others. At this point, it was determined that image quality or spermatozoa position within the image directly affected the predictive ability of the models.

In Figure 8, the rates at which the models detect the total and live/dead spermatozoa counts, based on spermatozoa density in the image, are shown. When examining YOLOv8, it was observed that it achieved successful detections in both images with low and high spermatozoa counts, and that the error margins were quite low. Similarly, the estimation of live/dead spermatozoa counts is also very close to the perfect estimate. YOLOv12 correctly detects more images containing both low and high spermatozoa counts. However, the error margins in images with errors are higher than those in YOLOv8. When it comes to dead/live predictions, it achieves a high prediction rate, comparable to YOLOv8, and even makes error-free predictions in images containing a large number of dead spermatozoa. YOLOv5 + HRNet, while producing acceptable predictions for visual spermatozoa density estimation, made errors in all dead spermatozoa counts when it came to dead/live predictions, despite correctly identifying the number of live spermatozoa. Examining Table 5, which presents the test-phase results, the fact that it incorrectly identified 786 of 1,008 dead spermatozoa cases indicates that YOLOv5 + HRNet is unable to detect dead spermatozoa accurately and often labels them as live. When examining the Faster R-CNN data, it was observed that the model made incorrect predictions in all images containing 10 or more spermatozoa, regardless of spermatozoa density. The dead/live prediction rate, however, was found to be acceptable.

In the mock test evaluation (confusion matrix in Table 8, performance metrics in Table 9), the expert evaluation served as the near-perfect reference standard, achieving a balanced accuracy of 98.8% (95% CI: 97.4–100.0%). Among the AI models, YOLOv12 demonstrated the highest performance with a balanced accuracy of 91.3% (95% CI: 87.9–94.8%) and an F1 score of 0.895. It showed strong specificity (93.6%) and precision (89.9%), correctly identifying 160 of 171 dead spermatozoa and 98 of 110 live spermatozoa.

**Table 8:** Confusion matrices for Expert, YOLOv8, YOLOv12, Faster R-CNN, and YOLOv5-HRNet models during mock tests for detecting live and dead spermatozoa.

Model	Class	Reference (ground truth)		Total
		Live	Death	
Expert	Live	108 (38.4%)	1 (0.4%)	
	Death	2 (0.7%)	170 (60.5%)	
	Total	110 (39.1%)	171 (60.9%)	281 (100%)
YOLOv8	Live	98 (34.9%)	16 (5.7%)	
	Death	12 (4.3%)	155 (55.1%)	
	Total	110 (39.1%)	171 (60.9%)	281 (100%)
YOLOv12	Live	98 (34.9%)	11 (3.9%)	
	Death	12 (4.3%)	160 (56.9%)	
	Total	110 (39.1%)	171 (60.9%)	281 (100%)
Faster R-CNN	Live	95 (33.8%)	13 (4.6%)	
	Death	15 (5.4%)	158 (56.2%)	
	Total	110 (39.1%)	171 (60.9%)	281 (100%)
YOLOv5 HRNet	Live	96 (34.2%)	36 (12.8%)	
	Death	14 (5.0%)	135 (48.0%)	
	Total	110 (39.1%)	171 (60.9%)	281 (100%)

**Table 9:** Performance comparison of YOLOv8, YOLOv12, Faster R-CNN, and YOLOv5-HRNet model mock sets in detecting live and dead spermatozoa.

Metrics	Models				
	Expert	YOLOv8	YOLOv12	Faster R-CNN	YOLOv5 HRNet
Recall	0.982 (0.936–0.998)	0.891 (0.817–0.942)	0.891 (0.817–0.942)	0.864 (0.785–0.922)	0.873 (0.796–0.929)
Specificity	0.994 (0.968–0.999)	0.906 (0.853–0.946)	0.936 (0.888–0.968)	0.924 (0.874–0.959)	0.790 (0.721–0.848)
Accuracy	0.989 (0.969–0.998)	0.900 (0.859–0.933)	0.918 (0.880–0.947)	0.900 (0.859–0.933)	0.822 (0.772–0.865)
Balanced accuracy	0.988 (0.974–1.000)	0.899 (0.862–0.935)	0.913 (0.879–0.948)	0.894 (0.856–0.932)	0.831 (0.787–0.875)
Precision	0.991 (0.950–0.999)	0.860 (0.782–0.918)	0.899 (0.827–0.949)	0.880 (0.803–0.934)	0.727 (0.643–0.801)
NPV	0.988 (0.959–0.998)	0.928 (0.878–0.862)	0.930 (0.881–0.963)	0.913 (0.861–0.951)	0.906 (0.847–0.948)
F1 Score	0.986 (0.971–1.000)	0.875 (0.829–0.921)	0.895 (0.852–0.938)	0.872 (0.824–0.919)	0.793 (0.737–0.849)
AUC	0.988 (0.972–1.000)	0.899 (0.857–0.941)	0.913 (0.873–0.953)	0.894 (0.850–0.938)	0.831 (0.780–0.882)
Cohen’s Kappa	0.978 (0.952–1.000)	0.792 (0.719–0.865)	0.828 (0.761–0.895)	0.790 (0.717–0.864)	0.640 (0.550–0.729)
Youden Index	0.976 (0.949–1.000)	0.797 (0.725–0.870)	0.827 (0.758–0.896)	0.788 (0.712–0.863)	0.662 (0.575–0.750)
McNemar’s test	*χ*^2^ = 0.13, df = 1, *p* = 0.564	*χ*^2^ = 0.57, df = 1, *p* = 0.449	*χ*^2^ = 0.04, df = 1, *p* = 0.835	*χ*^2^ = 0.14, df = 1, *p* = 0.706	*χ*^2^ = 9.68, df = 1, *p* < 0.01

NPV, negative predictive value; AUC, area under the curve; *χ*^2^, Chi-Square; df, degrees of freedom; *p*, *p*-value.

YOLOv8 performed similarly, with a balanced accuracy of 89.9% (95% CI: 86.2–93.5%) and an F1 score of 0.875. Faster R-CNN lagged slightly behind with a balanced accuracy of 89.4% (95% CI: 85.6–93.2%) and an F1 score of 0.872. YOLOv5 + HRNet again showed the lowest performance, with a balanced accuracy of 83.1% (95% CI: 78.7–87.5%), driven primarily by poor specificity (79.0%) and a higher number of false negatives in dead sperm detection.

To statistically compare the models’ discriminative ability, we performed DeLong’s test on the Area Under the Curve (AUC) values. The expert evaluator’s AUC (0.988) was significantly superior to all AI models (*p* < 0.001 for all comparisons), confirming its role as a robust reference standard. Among the automated models, YOLOv12 achieved the highest AUC (0.913), which was significantly better than both YOLOv8 (AUC: 0.899; *Z* = −2.26, *p* = 0.024) and Faster R-CNN (AUC: 0.894; *Z* = 2.21, *p* = 0.027). No significant difference was observed between YOLOv8 and Faster R-CNN (*Z* = 0.52, *p* = 0.600), indicating comparable overall performance. YOLOv5 + HRNet exhibited the lowest AUC (0.831) and was significantly inferior to all other models (*p* < 0.001 for all comparisons).

Youden’s Index results mirrored this performance trend. YOLOv12 again led the AI models (0.827), followed closely by YOLOv8 (0.797) and Faster R-CNN (0.788). The expert evaluator’s near-perfect index (0.976) validates the reference standard. In contrast, YOLOv5 + HRNet’s substantially lower index (0.662) quantifies its persistent weakness in balanced classification.

Statistical analysis using McNemar’s test indicated no significant difference between the predictions of YOLOv8, YOLOv12, or Faster R-CNN and the ground truth (*p* > 0.05). However, YOLOv5 + HRNet showed a statistically significant difference from the ground truth (*p* < 0.01), further confirming its inferior performance. The DeLong test results align with these findings, providing additional evidence that YOLOv12 significantly outperforms other AI models in overall discriminative ability, while YOLOv8 and Faster R-CNN demonstrate statistically equivalent performance.

## 4 Discussion

The current study aimed to measure bull semen viability with high reliability and in a manner as easy to apply as the Eosin/Nigrosin staining method, without requiring the expense and specialized expertise of flow cytometric analysis. To this end, four different artificial intelligence models were trained with a total of 3,068 images and then validated and tested.

The test set results (Table 7) showed that the YOLOv8 model achieved the highest overall performance, with balanced accuracy of 97.1% (95% CI: 96.4–97.7%), recall of 97.0%, and an F1 score of 0.978. YOLOv12 also demonstrated strong, comparable performance, with balanced accuracy of 94.1% (95% CI: 93.2–95.0%) and an F1 score of 0.958. In contrast, the two-stage Faster R-CNN architecture exhibited a significant decline in performance (balanced accuracy: 86.4%; 95% CI: 85.1–87.8%), characterized by higher false-positive (FP) and false-negative (FN) rates. The YOLOv5 + HRNet model performed the poorest, with a critically low specificity of 22.0% (95% CI: 19.5–24.7%) and a balanced accuracy of only 57.3% (95% CI: 55.9–58.7%). This was primarily due to its severe deficiency in detecting dead spermatozoa (negative class), leading to the misclassification of many as live, as evidenced by the high FN count for dead sperm in the confusion matrix (Table 6).

The poor performance of the YOLOv5 + HRNet combination is attributed to its severe failure in detecting dead spermatozoa (the negative class). The model’s low balanced accuracy of 57.3% (95% CI: 55.9–58.7) and critically low specificity of 22.0% (95% CI: 19.5–24.7) indicate a substantial misclassification of dead sperm (Table 7). This outcome may be related to the HRNet architecture’s strategy of preserving high-resolution feature maps. While Sun et al. (31) note that this architecture aims to produce representations that are both semantically strong and spatially sensitive, the U-HRNet study demonstrates that low-resolution feature maps generally yield stronger semantic representations, whereas high-resolution maps better characterize local features, such as edges, but contain weaker semantic information (38). In the present study, it is hypothesized that HRNet’s high-resolution preservation strategy may not have provided sufficient semantic richness to distinguish the subtle color differences between dead and live spermatozoa resulting from eosin/nigrosin staining. This limitation, combined with the class imbalance in the training set (low proportion of dead sperm), likely hindered the model’s ability to effectively learn the minority class. The model’s F1 score for dead sperm detection was substantially lower than that of the other architectures evaluated, further highlighting its deficiency in accurately identifying this class.

In the second phase of the study, a mock test was conducted to observe the real-world counterpart of the data obtained. During the mock test, a total of 281 spermatozoa were counted simultaneously by an expert and artificial intelligence models across 36 images. It is a natural outcome that the artificial intelligence models were significantly faster than the expert. However, when the estimated total spermatozoa data was examined, it was seen that YOLOv8 and YOLOv12 obtained data very close to perfect prediction. Although YOLOv5 + HRNet made highly accurate predictions, similar to YOLOv8 and YOLOv12, the number of incorrectly predicted spermatozoa in the images it mispredicted was quite high. In the Faster R-CNN case, errors were observed in all images containing 10 or more spermatozoa. At this point, Faster R-CNN will also achieve a usable level of accuracy in detecting images containing a small number of spermatozoa.

In terms of accurately detecting dead and live spermatozoa, both YOLOv8 and YOLOv12 achieved successful results; however, YOLOv12 performed better than YOLOv8 in images containing a large number of spermatozoa. While YOLOv5 + HRNet demonstrated extremely high performance in live spermatozoa count, it proved unusable because it completely misidentified dead spermatozoa. Faster R-CNN yielded results similar to YOLOv8 in predicting dead and live spermatozoa.

When examining the mock test’s success rate, unlike the test data, the most successful model was YOLOv12 (F1 0.895). YOLOv12 and YOLOv8 also yielded successful results, but the success level of Faster R-CNN and YOLOv5 + HRNet was not acceptable.

The Faster R-CNN model’s high FP and FN rates may stem from design constraints in its fundamental components. Firstly, the Region Proposal Network (RPN) stage, as defined in Ren et al. (27) original Faster R-CNN paper, employs a binary classification system (object/background) at each anchor position. During training, anchors with IoU > 0.7 were labeled as positive, while those with IoU < 0.3 were labeled as negative. It is considered that ambiguous examples falling between these thresholds may have contributed to missed detections in dense populations of spermatozoa. Secondly, the 32 × downsampling rate applied by the ResNet-50 backbone may have made it difficult to detect small objects. As noted in Lin et al. (39), deeper layers capture more semantic features but lose spatial resolution. For a typical 640 × 480 pixel input image, the final feature map is reduced to only 20 × 15 pixels in size. In this case, small spermatozoa heads may occupy less than 1 pixel, which may have negatively affected detection success. Thirdly, performance issues with the Non-Maximum Suppression (NMS) mechanism on overlapping objects have been reported by Bodla et al. (40) as undetectable when an object remains within a predefined overlap threshold. It is thought that this situation may lead to the systematic omission of spermatozoa that are close to each other or overlapping in microscopic preparations. In the present study, it is considered that spermatozoa frequently overlap in high-density preparations and that this situation may have suppressed the accurate detections of Faster R-CNN’s NMS mechanism.

When examining images where all models made errors, the causes of the errors were:

- The tail of the spermatozoon passing over the head.
- The presence of lighter or darker areas around the spermatozoon.
- Air bubbles being mistaken for spermatozoa.
- The head of the spermatozoon appears dark due to smear, even if it does not take up dye.
- Images that are not sufficiently clear cannot be detected.
- Including spermatozoa with detached heads in the count due to the analysis being performed only on the head, but some models do not recognize detached heads as spermatozoa.
- Spermatozoa overlapping.
- Spermatozoa that cannot be seen for no apparent reason.

**Figure 9:**
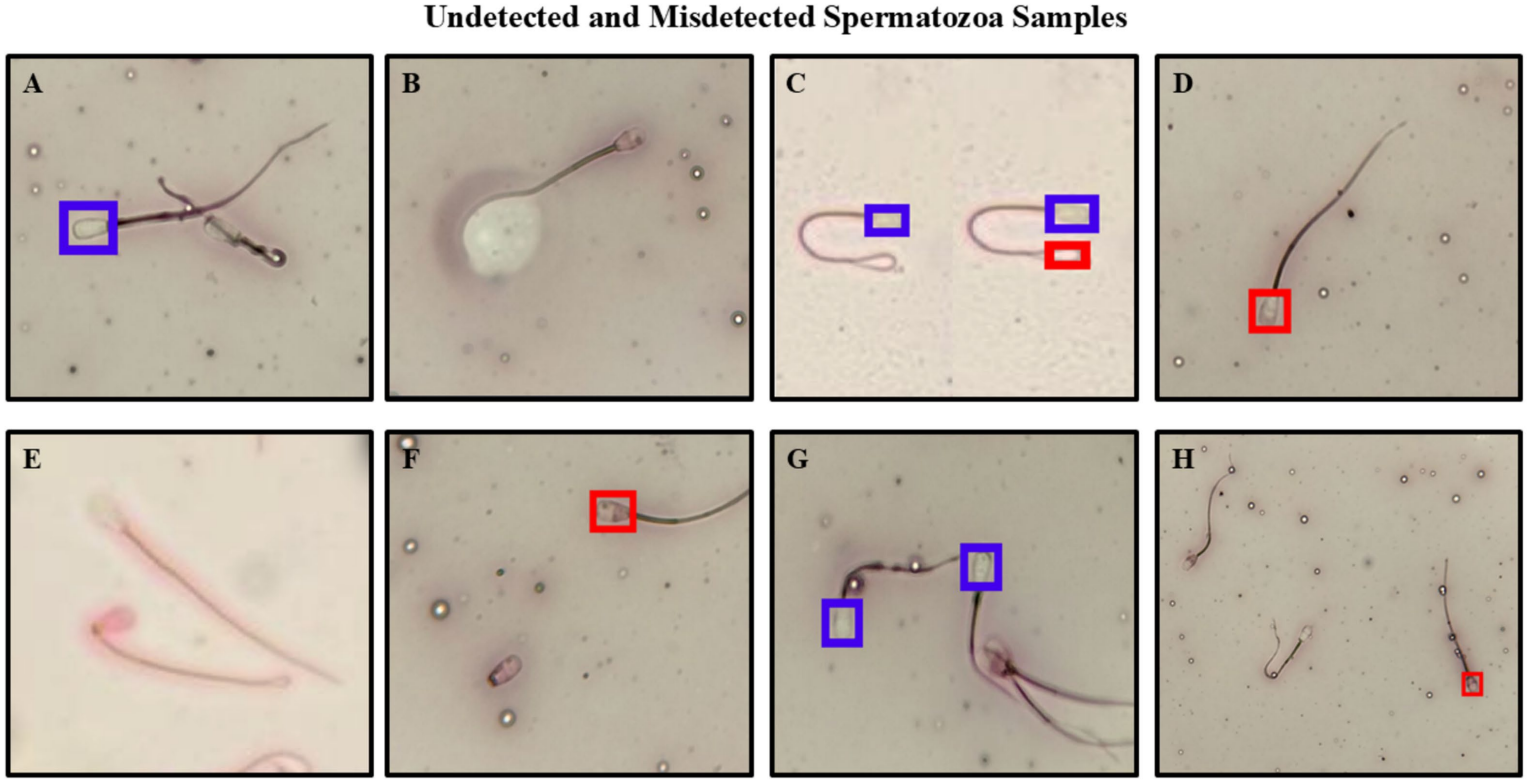
Spermatozoa that could not be detected or were misidentified for various reasons. Spermatozoon with tail passing over head **(A)**, spermatozoon with color change around it due to frottage **(B)**, spermatozoon with tail curved like head **(C)**, spermatozoon stained dark despite not taking up dye **(D)**, spermatozoa that could not be detected due to poor image quality **(E)**, spermatozoon with a broken head **(F)**, overlapping spermatozoa **(G)**, spermatozoa that could not be detected for no apparent reason **(H)**.

Have been identified (Figure 9).

Preparations prepared using the eosin/nigrosin method are counted visually, and spermatozoa that can be seen or distinguished by the human eye might not be detected by artificial intelligence. However, given the findings, the fact that new-generation artificial intelligence models yield better results than older generations will provide significant convenience for performing subjectivity-dependent analyses.

It is also quite important to address the strengths and limitations of the study. One of the study’s key strengths is that it is based on a large, meticulously annotated dataset comprising 27.525 labeled sperm heads from 3.068 microscopic images. Ground truth reliability has been quantitatively verified using IoU and Fleiss’ Kappa statistics, demonstrating high observer agreement. Model performance was evaluated not only using object-detection metrics but also through count-based analyses and an independent mock test simulating a routine laboratory workflow. Comparing different architectures under the same experimental conditions has provided the study with methodological transparency and technical robustness.

However, certain limitations should also be considered. All images were obtained in a single laboratory setting using a standardized microscope, imaging system, and staining protocol. While this controlled approach ensures methodological consistency, it may limit generalizability across different laboratory conditions, microscope systems, or staining protocols. Furthermore, although the mock test increases clinical interpretability, it does not constitute a multi-center, independent external validation. Therefore, extensive validation studies conducted in different laboratory environments are important for establishing the external validity of the system. In addition, while the YOLOv12 model has demonstrated strong performance, it has not yet been published in a peer-reviewed journal and is currently in the preprint stage. Future architectural changes, application improvements, or parameter updates may affect performance stability. Therefore, the reproducibility of the reported results depends on the specific version and configuration used in the study, and future updates may yield different results.

## 5 Conclusion

The present study demonstrates that artificial intelligence–based analysis of bull sperm viability is feasible and yields high diagnostic performance under controlled laboratory conditions. The results indicate that modern object detection architectures, particularly YOLOv8 and YOLOv12, can provide reliable and rapid viability assessments from eosin–nigrosin–stained smears. Nevertheless, the current system requires standardized smear preparation, consistent imaging conditions, and controlled sperm density within microscopic fields to maintain performance stability. Therefore, further validation across different laboratories, imaging devices, and staining environments is necessary to determine its generalizability. Given the subjectivity of manual counting methods and the cost and technical requirements of flow cytometry, artificial intelligence–assisted evaluation is a promising alternative. Ongoing studies are expanding this framework to assess additional advanced spermatological parameters.

## Data Availability

The original contributions presented in the study are included in the article/supplementary material, further inquiries can be directed to the corresponding author.
